# Analysis of the importance of using intermediate intervals in the calculation of geometrical characteristics of the ship’s hull

**DOI:** 10.1371/journal.pone.0348523

**Published:** 2026-05-08

**Authors:** Paweł Chorab, Dorota Łozowicka

**Affiliations:** Faculty of Navigation, Maritime University of Szczecin, Szczecin, Poland; IGDTUW: Indira Gandhi Delhi Technical University for Women, INDIA

## Abstract

This article examines the impact of incorporating intermediate sections in geometric hull calculations and highlights their role in improving the accuracy of underwater volume estimation and the determination of the center of buoyancy. The study demonstrates that neglecting intermediate sections can lead to substantial numerical errors, which may affect the reliability of hydrostatic parameters used in stability assessment. In the context of hydrodynamic analyses, accurate geometric representation remains essential for obtaining consistent and reproducible results. The presented results quantitatively assess the reduction of numerical integration error achieved by introducing intermediate sections and by comparing selected classical numerical integration schemes. Based on these findings, a genetic algorithm–based optimization framework is proposed as future work to support the automated selection of the number and spatial distribution of intermediate sections, as well as the integration strategy, while balancing accuracy and computational cost. The proposed framework is intended to provide a systematic approach for adapting hull discretization to local geometric characteristics and to support reproducible hydrostatic calculations in engineering applications.

## Introduction

Accurate determination of the geometric and hydrostatic characteristics of a ship hull is a fundamental element of design analyses, operational assessments, and safety evaluations of marine vessels. Parameters such as the submerged volume, the position of the center of buoyancy, and waterplane characteristics are directly used in stability calculations, and inaccuracies in their estimation may lead to significant errors in predicting vessel behavior under operational conditions.

In engineering practice, hydrostatic calculations are most commonly performed using approximate numerical methods based on discretizing the hull into a finite number of transverse sections, waterplanes, or longitudinal sections. The accuracy of these methods depends both on the adopted numerical integration scheme and on the discretization strategy. In particular, the inclusion of intermediate sections plays a crucial role in regions characterized by strong geometric variability, such as the bow and stern, where hull curvature changes in a nonlinear manner.

Accurate geometric representation of a ship hull is a prerequisite for reliable hydrostatic and hydrodynamic calculations. In particular, CAD-based hull modeling involves nontrivial geometric operations such as surface intersections, which may affect the robustness and consistency of downstream numerical evaluations [1]. Consequently, even at the stage of geometric model preparation, the adopted approximation and discretization strategies may significantly influence the final results of hydrostatic computations.

Similar issues related to geometric irregularity and model fidelity can also be observed in other branches of engineering, where computational results strongly depend on discretization strategies and the representation of asymmetric or irregular shapes, for example in seismic analyses of irregular structures [2]. This indicates that the problem of selecting an appropriate number and distribution of computational subdivisions is of a general nature and not limited exclusively to naval architecture.

Errors in the estimation of the submerged volume and the position of the center of buoyancy have been identified as contributing factors in several major maritime accidents, in which—despite compliance with formal stability criteria—vessels or cargo were lost. With the development of second-generation intact stability criteria, the importance of accurate geometric data has further increased, as hydrostatic parameters must now be evaluated under non-equilibrium conditions, such as when the hull is located on a wave crest or in a wave trough.

The sensitivity of stability-related performance to hull-form characteristics has been highlighted in the literature, including studies demonstrating that systematic variations of hull geometry can influence stability behavior under realistic operating conditions. This reinforces the need for accurate geometric and hydrostatic characterization as a basis for safety-relevant assessments [3]. Consequently, even relatively small geometric inaccuracies may, under certain conditions, result in noticeable differences in the vessel’s stability response.

The role of hull discretization and geometric representation has been addressed in a broad range of studies spanning both classical and modern approaches in naval architecture. Early observations by Froude highlighted the importance of increasing sectional density in regions of pronounced hull curvature to improve the accuracy of buoyancy-related predictions. More recent works have examined the influence of modeling and numerical techniques on hydrostatic and hydrodynamic computations, including finite element–based structural modeling [4], curve-fitting and surface representation methods affecting hydrostatic properties [5,6], and data-driven approaches for predicting hydrostatic characteristics [7]. Empirical and regression-based studies have further demonstrated the strong dependence of displacement and buoyancy-related parameters on hull geometry at the preliminary design stage [8]. Classical numerical procedures for hydrostatic calculations based on trapezoidal and Simpson-type integration remain widely applied in engineering practice [9], while comprehensive reviews continue to emphasize the central role of accurate geometric modeling in ship hydrostatics and stability assessments [10].

Previous studies have primarily focused on the selection of numerical integration schemes or on modifications of hull geometry in the context of resistance and hydrodynamic performance [11-14]. Considerably less attention has been devoted to a quantitative assessment of the influence of the number and spatial distribution of intermediate sections on the accuracy of classical hydrostatic calculations, particularly with respect to errors in submerged volume and center-of-buoyancy coordinates.

Previous studies have demonstrated the applicability of genetic algorithms and related optimization techniques in various ship design and analysis problems, including numerical parameter optimization [15], hull-form optimization coupled with potential-flow solvers and artificial intelligence [16], derivation of optimal hull geometries using evolutionary methods [17,18], and structural optimization of ship hulls [19-21]. In addition, optimization-oriented studies addressing hydrodynamic performance of bulk carriers further illustrate the relevance of such approaches in naval architecture [22]. However, the direct application of these methods to the optimization of intermediate section placement in classical hydrostatic calculations remains largely unexplored.

The aim of this study is to quantitatively assess the influence of including intermediate sections on the accuracy of geometric and hydrostatic calculations of a ship hull, with particular emphasis on the submerged volume and the position of the center of buoyancy. The analysis is conducted using a real vessel as a case study and employs classical numerical integration methods. In addition, a conceptual framework for the application of genetic algorithms to optimize the number and distribution of intermediate sections is proposed and explicitly treated as a direction for future research, rather than as a method implemented and validated within the present study.

## Materials and methods

### Geometric variables and hydrostatic parameters

In situations where the ship’s hull is in an arbitrary position relative to the water surface plane, the submerged part of the hull is characterized by a set of geometric and hydrostatic parameters. These parameters form the basis for stability and hydrostatic analyses and are defined as follows:

- Submerged volume *V* [m³],
- Coordinates of the center of buoyancy *B*: *X*_*B*_*, Y*_*B*_*, Z*_*B*_ [m],
- Waterplane area *A*_*W*_ [m²],
- Coordinates of the centroid of the waterplane area, referred to as the center of flotation: *X*_*F*_*, Y*_*F*_*,*[m]
- Moments of inertia of the waterplane area with respect to axes passing through the center of flotation: longitudinal moment of inertia: *I*_*L*_, transverse moment of inertia: *I*_*T*_.[m^4^]

Quantities such as the submerged volume V and the coordinates of the center of buoyancy can be determined using:

- Transverse waterplane sections of the hull (for heel angle *Φ* = 0° and trim angle *Θ* = 0°),
- Transverse frame (station) sections of the hull (for *Φ* = 0° and *Θ* ≠ 0°),
- Longitudinal sections of the hull (for *Φ* ≠ 0° and *Θ* = 0°).

where:

- *Φ* denotes the heel angle of the hull relative to the water surface plane [°],
- *Θ* denotes the trim angle of the hull relative to the water surface plane [°].

### Geometric characteristics of a ship hull using waterplane, buttock, and station methods

#### Waterplane section method.

This method is primarily used when the vessel is in an upright position, i.e., with no trim and no heel. When transverse waterplane sections are known, the submerged volume of the hull and the coordinates of the center of buoyancy for a given draft (*T*_*i*_), can be determined using the following input data:

- Waterplane areas *A*_*W*_, as a function of draft *f(T*_*i*_*),*
- m_WY_ – static moment of the waterplane area with respect to the intersection line between the waterplane section and the baseline plane; the moment is evaluated as a function of the local draft f(T_i_).

The submerged volume, the center of buoyancy coordinates, as well as the static moments of the submerged volume:

- *M*_*YZ*_ is calculated relative to the center plane,
- *M*_*XY*_ is calculated relative to the base plane,
- X_B_, Z_B_ are coordinates of the center of buoyancy B [m],

are computed as follows:


$$V=\int_{Z_{A}}^{Z=T_{i}}A_{W}(z)dz$$ (1)

$$M_{yz}=\int_{Z_{A}}^{Z=T_{i}}m_{Wy}(z)dz$$ (2)

$$M_{xy}=\int_{Z_{A}}^{Z=T_{i}}A_{W}(z)zdz$$ (3)

$$x_{B}=\frac{M_{yz}(z)}{V(z)}$$ (4)

$$z_{B}=\frac{M_{xy}(z)}{V(z)}$$ (5)

#### Buttock section method.

This method is applied to ships with trim but without heel. Buttock sections are used for the calculations. The geometric characteristics of the submerged part of the hull, cut off by the calm water surface inclined at an angle *Θ* to the baseline, will be determined. To calculate the submerged volume and coordinates of the center of buoyancy for a given draft, it is necessary to know:

- The areas of the buttock sections *A*_*S*_ as a function of *T*_*i*_,
- The static moments of these areas relative to the edge of intersection of the buttock section planes with the baseline *m*_*SY*_ as a function of *T*_*i*_.

It should be noted that the draft must be determined considering the trim angle as follows:


$$T_{i}=T+x_{i}\cdot tg\theta$$ (6)

The area and static moment are calculated for the given buttock section as a function of the draft.

The submerged volume, coordinates of the center of buoyancy (coordinates are defined in the subsequent paragraphs) and static moments of the submerged volume are calculated as follows:


$$\text{V}=\int_{L\text{0}}A_{S}\left(x,T_{i}\right)dx=\text{V}(T,\theta )$$ (7)

$$M_{yz}=\int_{L\text{0}}A_{s}\left(x,T_{i}\right)xdx=M_{yz}(T,\theta )$$ (8)

$$M_{xy}=\int_{L\text{0}}m_{Sy}\left(x,T_{i}\right)xdx=M_{xy}(T,\theta )$$ (9)

Knowing the displacement volume and its static moments relative to the coordinate system planes, the coordinates of the center of buoyancy can be easily calculated:


$$X_{B}=\frac{M_{yz}}{\text{V}}=\frac{\int_{L\text{0}}A_{S}(x,T_{i)}xdx}{\int_{L\text{0}}A_{S}(x,T_{i)}dx}=X_{B}(T,\;\theta )$$ (10)

$$Z_{B}=\frac{M_{xy}}{\text{V}}=\frac{\int_{L\text{0}}m_{S}(x,T_{i)}xdx}{\int_{L\text{0}}A_{S}(x,T_{i)}dx}=Z_{B}(T,\;\theta )$$ (11)

The integrals used in the formulas are evaluated numerically, e.g., using Simpson’s rule.

#### Longitudinal (station) section method.

Longitudinal sections can be effectively used to determine the displacement and coordinates of the center of buoyancy for a heeled but not trimmed ship. To perform these calculations, the following data are required:

- the areas of longitudinal sections *A*_*B*_,
- the static moments of these areas with respect to the edge of intersection between the longitudinal section planes and the baseline plane *m*_*BX*_.

These quantities are functions of the immersion of the longitudinal section and its distance from the plane of symmetry. Knowing the areas and their static moments for a given heeled waterplane, the displacement *V* and the coordinates of the center of buoyancy can be easily determined:


$$\text{V }=\;\int_{B_{\Phi }}A_{B}(y,T_{k})dy=\text{V}(T,\;\Phi )$$ (12)

$${\text{M}}_{xy}\;=\;\int_{B_{\Phi }}m_{Bx}(y,T_{k})dy={\text{M}}_{xy}(T,\;\Phi )$$ (13)

$${\text{M}}_{xz}\;=\;\int_{B_{\Phi }}A_{B}(y,T_{k})ydy={\text{M}}_{xz}(T,\;\Phi )$$ (14)

$$Y_{B}=\frac{M_{xz}}{\text{V}}=\frac{\int_{B\Phi }A_{B}(y,T_{k})ydy}{\int_{B\text{0}}A_{B}(y,T_{k})dy}=Y_{B}(T,\;\Phi )$$ (15)

$$Z_{B}=\frac{M_{xy}}{\text{V}}=\frac{\int_{B\Phi }m_{Bx}(y,T_{k})dy}{\int_{B\text{0}}A_{B}(y,T_{k})dy}=Z_{B}(T,\;\Phi )$$ (16)

The integrals are evaluated numerically using methods such as Simpson’s rule. Calculation accuracy is improved by increasing the number of longitudinal sections, which also enables determination of the longitudinal coordinate of the center of buoyancy through the evaluation of the corresponding static moment with respect to the intersection of the section plane and the baseline.

### Numerical integration methods

Numerical methods are preferred over analytical solutions when the function is too complex or impossible to integrate exactly. They provide approximate values of definite integrals, with accuracy depending on the chosen method. In general, these methods involve dividing the integration domain into smaller subregions where the area can be calculated. Summing the areas of these regions gives an approximate value of the integral. Greater accuracy is obtained by increasing the number of segments (subintervals). The final result is the sum of the individual subareas under the curve within the specified interval. To illustrate the accuracy of numerical methods, an example as shown in Eq. (A1) in Appendix A from the work by Kwaśniewski [23] is used, which involves calculating the area under the curve of a known integrable function. The detailed computational example is provided in the Appendix A in S1 File.

### The impact of using intermediate stations on errors in the calculation of ship hull geometrical characteristics

In the course of the research conducted by the authors, various approximate integration methods were selected and the accuracy of each method was quantitatively assessed. Preliminary results of this investigation were presented in the study by Kwaśniewski [23]. Among other findings, the accuracy of the obtained values for selected geometrical parameters was evaluated by comparing them with values extracted from the vessel’s geometric characteristics.

The geometric and hydrostatic calculations presented in this study were carried out using the design documentation of a real merchant vessel, m/v Mazowsze, operated by the Polish Steamship Company. The hull geometry was defined on the basis of the ship’s lines plan and hydrostatic documentation, which include transverse stations, waterlines, buttock lines, and associated geometric characteristics required for classical hydrostatic calculations.

This section presents the computational procedures and the algorithm used to determine the geometrical characteristics for a selected waterline, using the ordinates method.

The station spacing value *dx* was determined by dividing the total length of the examined vessel (183 meters) by the number of intervals (20), yielding *dx = 9,15* [m]. For intermediate stations, the value of dx is halved, resulting in ½ dx = 4,575 [m]. The distances from the aft perpendicular to the end points along each waterline were read from the vessel’s lines plan (side elevation view).

The 8-meter draft waterline does not intersect the main transverse sections (stations), necessitating the inclusion of additional intermediate stations labeled 0’, 0.5’, 19.5” and 20.” These stations are spaced at adjusted intervals *dx’, dx,”* with associated correction coefficients *λ’* and *λ,”* respectively. The positions of certain ordinates, such as X_OW_ 0.5’ and 19’, also require correction. In this specific example, the application of correction coefficients for stations 0’ and 20’ is unnecessary, as their contributions are nullified by a multiplication with zero—rendering the result invariant regardless of the correction factor used.

The corrected segment lengths *d* were calculated as follows:

- The starting point of the waterline, station 0’, measured from the aft perpendicular is 2,2 [m],
- The endpoint of the waterline, station 20’, measured from the aft perpendicular is 187 [m],
- Distance from the aft perpendicular to station no 1 is 9,15 [m],
- Distance from the aft perpendicular to station no 19 is 173,85 [m].

From these values, the adjusted segment lengths were determined as:

- *dx’* = 3.475 [m]
- *dx” =* 6.575[m]

Subsequently, Simpson’s correction coefficients *W*_*S*_ [-] were computed for the intermediate stations 0’; 0,5’; 19,5’ and 20’. The following values were obtained:

- *λ’* = 0.7596 [-],
- *λ”* = 1.437 [-].

Based on these calculations, the Simpson coefficients were corrected for stations 0’; 0,5’; 1; 19; 19,5’, and 20’.

The distances from the aft perpendicular to stations 0,5’ and 19,5’ were determined to be 5,675 [m] and 180,425 [m], respectively. These distances were used to interpolate, via linear interpolation, the values of:

- cross-sectional areas of each station *A* [m²],
- first moments of area about the baseline *M*_*Z*_ [m²·m],
- absolute distances from the moulded baseline *X*_*OW*_ [m].

The computed results for each parameter, using Simpson’s method for the 8-meter draft waterline, are presented in Table 1. These data served as the basis for calculating selected values of the hull’s geometrical characteristics.

**Table 1:** Tabulated Simpson’s rule for approximate integration using the station method for the 8-meter waterline of the m/v Mazowsze.

Frame number	A	M_Z_	W_S_	X_OW_	Ws ∙ a	M_Z_ ∙ W_S_	A ∙ W_S_ ∙ X_OW_
**0’**	0	0	0,189891	−10	0	0	0
**0,5’**	10,68383	64,13907	0,759563	−9,380	8,115	48,72	−76,12
**1**	34,01	185,54	0,439891	−9	14,961	81,62	−134,65
**1,5**	67,01	366,4	1	−8,5	67,010	366,40	−569,59
**2**	100,84	528,62	0,5	−8	50,420	264,31	−403,36
**2,5**	129,08	644,24	1	−7,5	129,080	644,24	−968,10
**3**	153,05	729,13	0,75	−7	114,788	546,85	−803,51
**4**	187,92	834,41	2	−6	375,840	1668,82	−2255,04
**5**	209,87	885,92	1	−5	209,870	885,92	−1049,35
**6**	221,58	906,38	2	−4	443,160	1812,76	−1772,64
**7**	226,55	911,95	1	−3	226,550	911,95	−679,65
**8**	227,28	912,42	2	−2	454,560	1824,84	−909,12
**9**	227,28	912,42	1	−1	227,280	912,42	−227,28
**10**	227,28	912,42	2	0	454,560	1824,84	0,00
**11**	227,28	912,42	1	1	227,280	912,42	227,28
**12**	227,28	912,42	2	2	454,560	1824,84	909,12
**13**	227,28	912,42	1	3	227,280	912,42	681,84
**14**	227,28	912,42	2	4	454,560	1824,84	1818,24
**15**	226,68	912,07	1	5	226,680	912,07	1133,40
**16**	223,56	908,81	2	6	447,120	1817,62	2682,72
**17**	212,53	880,25	0,75	7	159,398	660,19	1115,78
**18**	184,05	780,14	0,5	8	92,025	390,07	736,20
**18,5**	160,56	688,56	1	8,5	160,560	688,56	1364,76
**19**	130,17	565,68	0,60929	9	79,311	344,66	713,80
**19,5’**	72,43891	326,3983	1,437158	9,719	104,106	469,09	1011,76
**20’**	0	0	0,35929	11,765	0,000	0,00	0

The station-based integration method was used to obtain the following hydrostatic parameters: underwater volume, vertical position of the center of buoyancy, longitudinal (horizontal) position of the center of buoyancy.

The underwater volume was determined by calculating the area under the curve representing the cross-sectional areas of transverse stations as a function of their distance from the aft perpendicular. This integration was performed using a selected approximate integration method.

The underwater volume was computed using Simpson’s rule, based on the data provided in Table 2. The resulting value is as follows:

**Table 2:** Tabulated Simpson’s rule for approximate integration using the station method for the 8-meter waterline of the m/v Mazowsze, excluding intermediate stations.

Frame number	A	W_S_	W_S_ ∙ A	MZ	X_OW_	MZ ∙ W_S_	A ∙ W_S_ ∙ X_OW_
**0’**	0	0,440	0	0	−10	0	0
**1’**	42,044	1,760	73,97906	226,785	−8,880	399,0425	−656,918
**2**	100,84	0,940	94,77858	528,62	−8	496,845	−758,229
**3**	153,05	2	306,1	729,13	−7	1458,26	−2142,7
**4**	187,92	1	187,92	834,41	−6	834,41	−1127,52
**5**	209,87	2	419,74	885,92	−5	1771,84	−2098,7
**6**	221,58	1	221,58	906,38	−4	906,38	−886,32
**7**	226,55	2	453,1	911,95	−3	1823,9	−1359,3
**8**	227,28	1	227,28	912,42	−2	912,42	−454,56
**9**	227,28	2	454,56	912,42	−1	1824,84	−454,56
**10**	227,28	1	227,28	912,42	0	912,42	0
**11**	227,28	2	454,56	912,42	1	1824,84	454,56
**12**	227,28	1	227,28	912,42	2	912,42	454,56
**13**	227,28	2	454,56	912,42	3	1824,84	1363,68
**14**	227,28	1	227,28	912,42	4	912,42	909,12
**15**	226,68	2	453,36	912,07	5	1824,14	2266,8
**16**	223,56	1	223,56	908,81	6	908,81	1341,36
**17**	212,53	2	425,06	880,25	7	1760,5	2975,42
**18**	184,05	1,1093	204,1648	780,14	8	865,4012	1633,318
**19’**	111,866	2,4372	272,6352	489,673	9,21858	1193,411	2513,309
**20’**	0	0,6093	0	0	10,25	0	0


$$V=\frac{\text{2}dx}{\text{3}}\cdot \Sigma \left(a\cdot W_{s\;}\right)$$ (17)

For comparison purposes, the area under the curve of transverse cross-sectional areas was also computed using the Midpoint Rule for numerical integration. In this method, the sectional areas are evaluated at midpoints of each of the twenty intervals defined along the distance from the aft perpendicular.

The result obtained using the Midpoint Rule is as follows:


$$V=dx\cdot (f(y_{\text{1}}{+y}_{\text{2}}+\ldots +y_{i})$$ (18)

For comparison, the area under the curve representing the cross-sectional areas of the ship’s transverse stations was also calculated using the Midpoint Rule for numerical integration. In this method, the sectional areas are evaluated at the midpoints of each of the twenty intervals along the longitudinal axis, i.e., halfway between the adjacent station positions measured from the aft perpendicular.

The result obtained using the Midpoint Rule is as follows:


$$V=dx\cdot (f(y_{\text{1}}{+y}_{\text{2}}+\ldots +y_{i})$$ (19)

In the next step, the coordinates of the center of buoyancy were calculated, including:


$$M_{xy}=\frac{\text{2}dx}{\text{3}}\Sigma (m_{z\cdot }W_{s})$$ (20)

$$M_{yz}=\frac{\text{2}dx^{\text{2}}}{\text{3}}\Sigma (a\cdot W_{s}\cdot x_{ow})$$ (21)

$$Z_{B}=\frac{M_{xy}}{V}$$ (22)

$$X_{B}=\frac{M_{yz}}{V}$$ (23)

An important consideration in the approximate integration of geometric characteristics is the inclusion of intermediate stations or waterlines at locations where the parameter curves exhibit the greatest variability. This typically occurs at the boundaries of waterlines or near the extremities of the submerged hull form. Although this increases the computational effort, it significantly improves the accuracy of the results.

### Conceptual framework for genetic algorithm–based optimization

Based on the findings of this study, a conceptual framework for applying genetic algorithms to the optimization of hull discretization is proposed as a direction for future research. The genetic algorithm is not implemented or validated in the present study.

The problem addressed by the authors concerns the selection of an optimal approximate integration method in order to minimize numerical error in the hydrodynamic analysis of a ship hull’s shape. Input parameters include both the hull geometry (e.g., bow shape, stern symmetry), the method used to determine underwater geometric characteristics (e.g., station-based, buttock-based, or waterline-based integration), and the type of numerical method applied.

Considering the review of existing applications of evolutionary methods—including genetic algorithms (GAs)—as well as their versatility and minimal requirements regarding the form of the objective function, the authors decided to employ this approach to address the posed optimization problem.

The choice of optimization method was primarily driven by the intended encoding of input parameters and the formulation of the objective function. Since analytical optimization methods require continuity and differentiability of the objective function, and cannot be applied to discrete problems, they were excluded from consideration. In the context of analyzing the geometric characteristics of a hull, the objective function may be complex and difficult to define analytically. Genetic algorithms are effective in such cases, as they operate based on fitness evaluation rather than relying on an explicit analytical formulation of the objective function.

GAs are well suited for exploring high-dimensional parameter spaces, which is crucial for identifying the optimal integration method when taking into account both hull geometry and the diversity of numerical integration techniques.

The problem under investigation requires multi-objective optimization, involving not only numerical accuracy but also computational time, algorithmic complexity, and other criteria. Genetic algorithms can be adapted for multi-objective optimization, enabling a balanced compromise between these competing aspects in the selection of the integration method.

A key advantage of genetic algorithms is their global search capability, which is essential when searching for an optimal integration method within a complex solution landscape typical of hydrodynamic analysis. GAs can be particularly effective when there is a need for automatic selection of integration parameters in response to specific hull shape characteristics and accuracy requirements.

This section presents the concept of using genetic algorithms to identify combinations of parameters (including the number and size of intermediate segments) that minimize numerical errors. Preliminary results have shown that the most significant factor influencing the accuracy of computed results is the numerical error associated with approximations made in calculating the longitudinal position of the center of buoyancy. Therefore, the genetic algorithm concept will be demonstrated using the example of optimizing the integration method for calculating this particular parameter.

The longitudinal position of the centre of buoyancy can be calculated as


$$X_{B}=\frac{\int_{v}x\cdot \rho (x,y,z)dV}{\int_{v}\rho (x,y,z)dV}$$ (24)

where:

- V is the volume of the submerged part of the hull,
- X is the horizontal coordinate (longitudinal axis),
- ρ(x,y,z) is the local fluid density (typically assumed constant in most practical applications).

This integral is commonly evaluated using approximate numerical integration methods (as discussed in the Materials and Methods section), which introduces numerical errors. The primary objective, therefore, is to minimize this error.

To optimize the computation of X_B_, we propose the application of genetic algorithms (GAs) to systematically select and tune the following:

1. The numerical integration method (e.g., Simpson’s first rule, trapezoidal rule),
2. The number of integration points (i.e., discretization resolution for the selected method),
3. The interpolation scheme used within the computational mesh,
4. Selected geometric parameters of the hull.

The goal of the GA-based optimization is to find the combination of these variables that results in the minimal deviation from a reference value of *X*_*B*_ obtained either analytically (if possible), from high-precision simulations, or experimental data.

**Fig 1:**
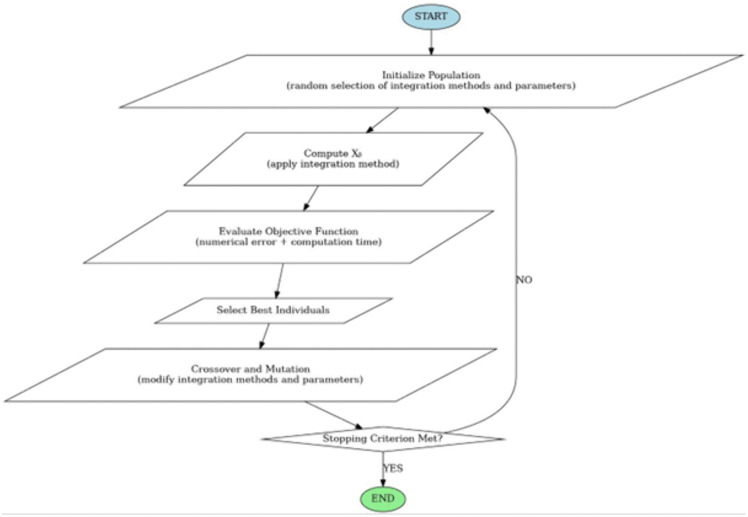
Flowchart of the Genetic Algorithm for optimizing the numerical integration of X_B._

The structure and workflow of the genetic algorithm used in the optimization process are illustrated in Fig 1 (see below).

The objective function can be defined as:


$$F=|\overset{reference}{\underset{B}{x}}-\overset{approx}{\underset{B}{x}}|+\lambda \cdot T\;$$ (25)

where:

- *X*_*B*_^*reference*^ is the reference value of the longitudinal centre of buoyancy (obtained analytically or through a high-precision method, e.g., a detailed CFD simulation),
- *X*_*B*_^*approx*^ is the value computed using an approximate method,
- *T* is the computation time,
- *λ* is a weighting coefficient used to balance accuracy and computational efficiency.

The representation of a solution within the genetic algorithm is a chromosome, which encodes a set of parameters such as the type of numerical integration method and the subdivision of the hull, including the number of intermediate segments. If the number of intermediate segments exceeds what is available in the source documentation, an interpolation method is selected to estimate the missing values. The initial population of chromosomes is generated randomly.

In the next step of the algorithm, the value of *X*_*B*_ is calculated for each chromosome. The objective function is then evaluated by computing the absolute error with respect to the reference value.

The genetic algorithm uses the following operators:

- Selection – e.g., tournament or roulette-wheel selection, used to identify and propagate the most promising integration methods and configurations,
- Crossover – random mixing of numerical integration methods and hull geometry parameters between selected parent chromosomes,
- Mutation – random alterations such as changing the number of integration points or switching to a different interpolation scheme.

Each individual (i.e., a combination of numerical method and associated parameters) is tested on a set of hydrodynamic scenarios. The algorithm terminates when improvements in accuracy become marginal or converge below a predefined threshold.

Genetic algorithms offer a powerful approach for minimizing numerical errors in ship hull hydrodynamic analysis by optimizing parameters of the approximate integration process. This methodology enables the automatic selection of numerical methods, number of segments, and interpolation strategies, resulting in improved computational accuracy while simultaneously optimizing resource usage and calculation time.

## Result and analysis

The geometric and hydrostatic characteristics of the m/v Mazowsze were analyzed based on the ship’s lines plan and hydrostatic documentation. Using the provided design data, key parameters such as transverse stations, waterlines, and buttock lines were examined, forming the basis for the subsequent hydrostatic calculations presented below.

To illustrate the impact of using intermediate subdivisions, the hydrostatic characteristics for the 8-meter waterline were also calculated using Simpson’s rule without including intermediate stations at positions 0,5; 1,5; 2,5; 17,5; 18,5, and 19,5. The results of the Simpson-based integration for the 8-meter waterline, excluding intermediate stations, are presented in Table 2.

Results of the geometric parameter calculations based on the data from Table 2 are as follows:


$$V=\frac{\text{2}dx}{\text{3}}\cdot \Sigma \left(a\cdot W_{s\;}\right)$$ (26)

$$M_{xy}=\frac{\text{2}dx}{\text{3}}\Sigma (m_{z\cdot }W_{s})$$ (27)

$$M_{yz}=\frac{\text{2}dx^{\text{2}}}{\text{3}}\Sigma (a\cdot W_{s}\cdot x_{ow})$$ (28)

$$Z_{B}=\frac{M_{xy}}{V}$$ (29)

$$X_{B}=\frac{M_{yz}}{V}$$ (30)

Table 3 summarizes the calculated values of selected geometric parameters using approximate integration methods, both with and without the inclusion of intermediate stations. This comparison aims to clearly demonstrate the advantages of incorporating intermediate frames. The results from both calculation approaches are compared against reference characteristic values obtained from the vessel’s documentation.

**Table 3:** Comparison of approximate integration results with and without the inclusion of intermediate frames.

	Reference value obtained from ship documentation	Results with the inclusion of intermediate frames			Results without the inclusion of intermediate frames		
		Value [m]	Difference V [m]	Error ε [%]	Value [m]	Difference V [m]	Error ε [%]
**V**	34230,8	34222,36	8,438855	0,0246589	34213,54313	−17,2569	−0,05041
**X**_**B**_	6,641	6,613722	−0,02728	−0,0278	6,481962191	0,159038	0,16205
**Z**_**B**_	4,17	4,169745	−0,00026	−0,00612	4,1667331	0,00382	0,09176

The reference values of the analysed geometric parameters of the ship’s hull were established on the basis of the Stability Booklet (m/v Mazowsze) approved by the classification society (Polish Register of Shipping).

**Fig 2:**
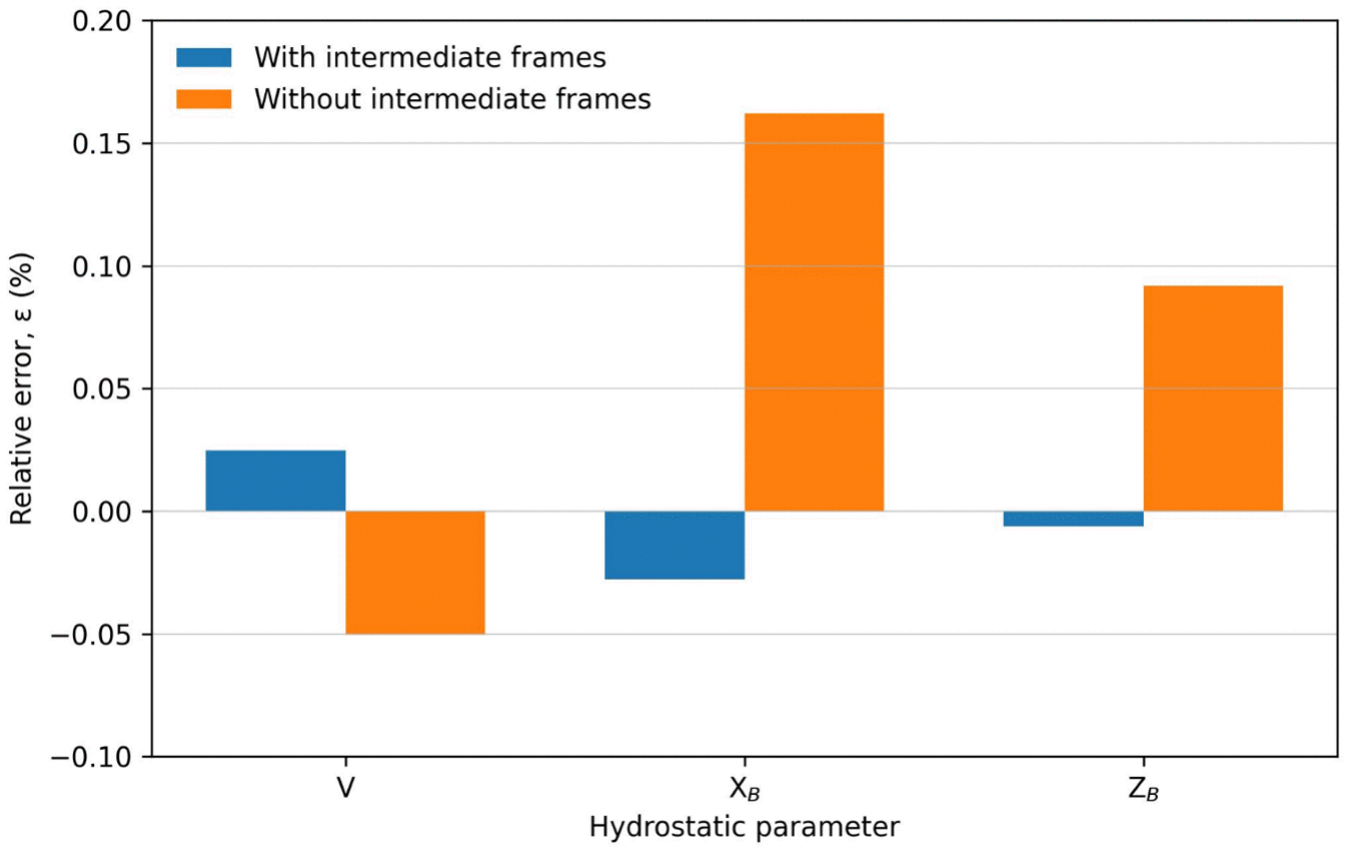
Relative errors ε [%] of selected hydrostatic parameters V, X_B_, and vertical Z_B_ calculated with and without the inclusion of intermediate frames, referenced to values obtained from ship documentation.

**Fig 3:**
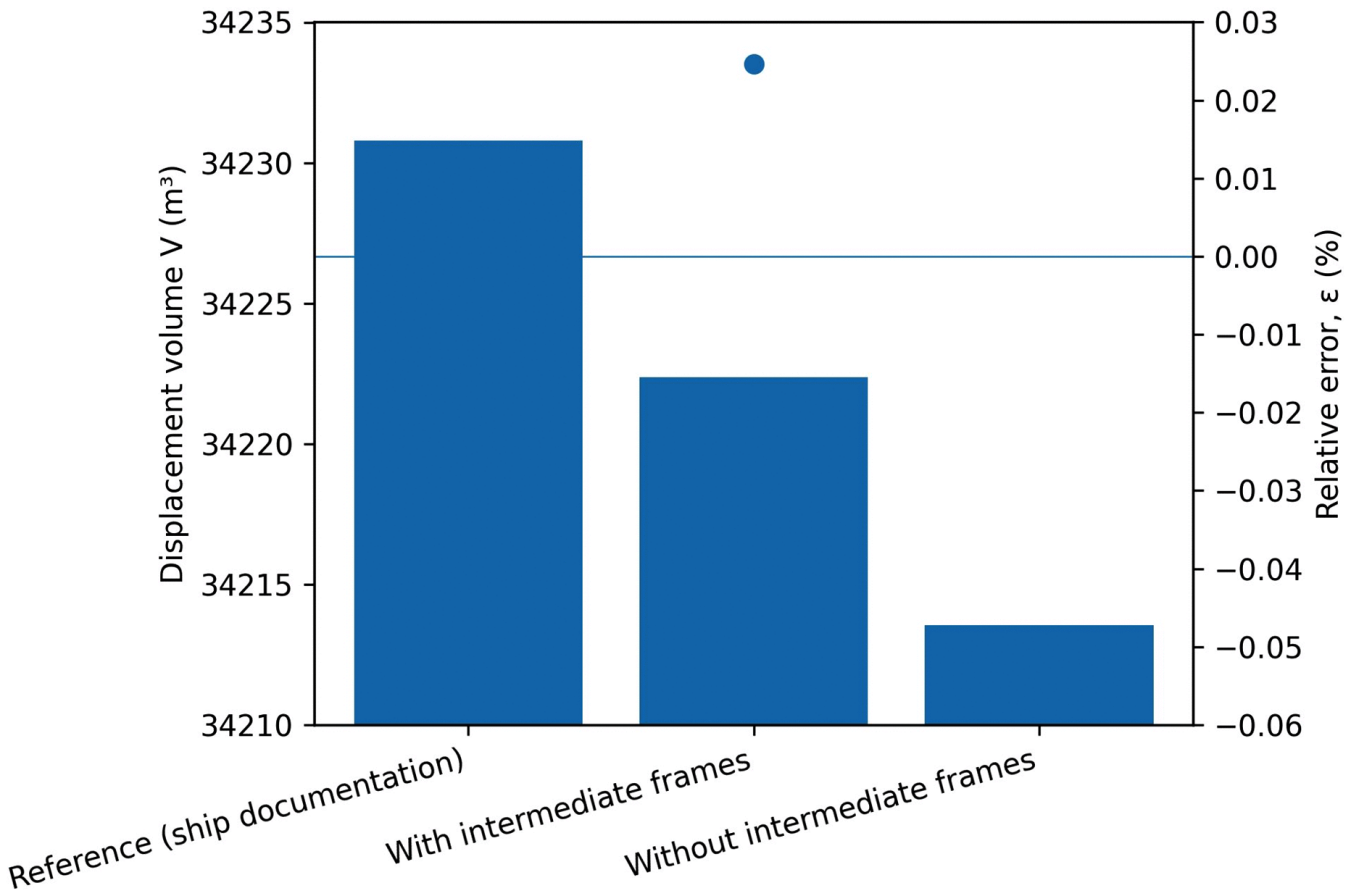
Displacement volume V obtained from ship documentation and numerical calculations performed with and without the inclusion of intermediate frames, together with the corresponding relative errors ε [%].

**Fig 4:**
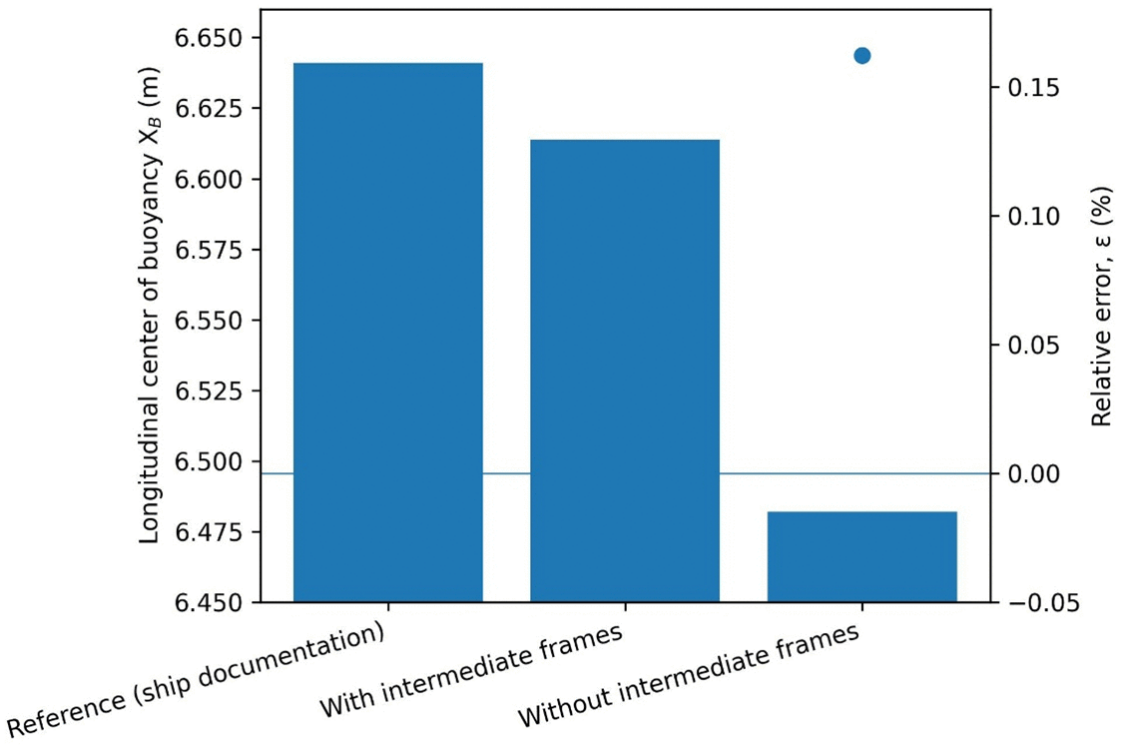
Longitudinal center of buoyancy X_B_ obtained from ship documentation and numerical calculations performed with and without the inclusion of intermediate frames, together with the corresponding relative errors ε [%].

**Fig 5:**
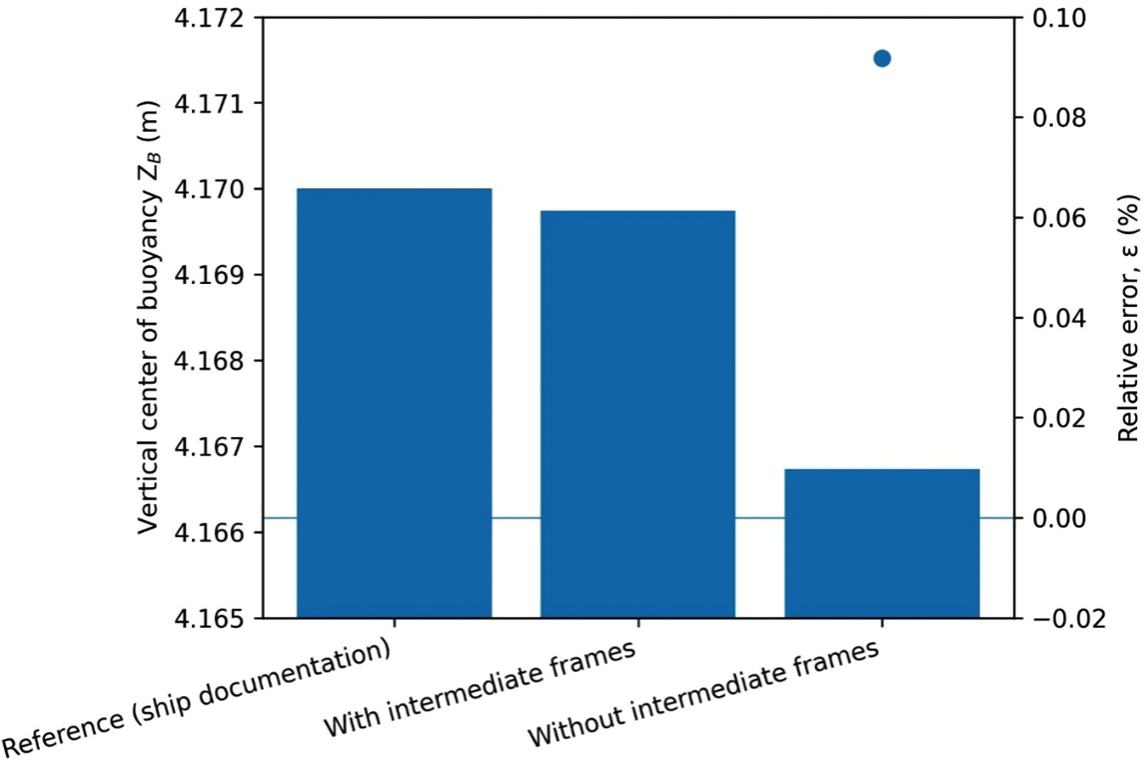
Vertical center of buoyancy Z_B_ obtained from ship documentation and numerical calculations performed with and without the inclusion of intermediate frames (bars, left axis), together with the corresponding relative errors ε [%].

Table 3 presents the deviations of the calculated results from the reference values, comparing cases with and without the inclusion of intermediate frames. The Value column shows the result for each geometric characteristic obtained via approximate integration. The Difference column displays the discrepancy between the reference value, derived from the hydrostatic curves of the m/v Mazowsze, and the calculated result obtained through numerical integration. The Error (%) column indicates the percentage error, representing the deviation of the calculated value from the reference, relative to the approximate integration result. A graphical comparison of the relative errors and corresponding hydrostatic parameters is presented in Figs 2–5.

Interpreting the data, it can be observed that although the percentage differences appear negligible, a clear increase in error is evident in each parameter when intermediate frames are omitted. This trend is clearly illustrated in Fig 2, which compares the relative errors of selected hydrostatic parameters calculated with and without intermediate frames, together with the corresponding displacement volume values. The smallest deviation, 0.05041 [%], was observed for the submerged volume parameter, corresponding to a difference of 17.26 [m³]. When intermediate frames were included, this deviation was reduced to 8.439 [m³], or only 0.025 [%], as shown in Fig 3.

The largest error in the presented example was associated with the longitudinal center of buoyancy position. In the case without intermediate divisions, an error of 0.162 [%] was observed, corresponding to a difference of nearly 16 [cm]. When intermediate frames were included, this difference was reduced to 2.73 [cm], representing only 0.0278 [%] deviation from the reference value, as illustrated in Fig 4.

A similar reduction in error can be observed for the vertical center of buoyancy, presented in Fig 5.

Despite the seemingly small magnitude of errors for both techniques, the use of intermediate frames reduced the percentage error by approximately a factor of five across all analyzed parameters.

### Example calculation of hydrostatic and stability parameters

In order to quantitatively assess the influence of using intermediate sections on the accuracy of calculations of the underwater volume and stability parameters, a numerical analysis was carried out using commercial ship design software **Maxsurf Modeler** and **Maxsurf Stability** (Bentley Systems). This software belongs to the standard tools used in ship design and stability analysis and applies numerical integration methods based on hull discretization by transverse sections (stations).

The hull models were defined as NURBS (Non-Uniform Rational B-Splines) surfaces, which ensure a continuous and smooth representation of hull geometry and allow controlled discretization of sections along the ship’s length. All calculations were performed for the same, unchanged hull geometry under identical draught conditions. The only variable in the analysis was the number and distribution of transverse sections (stations) used for numerical integration.

To increase the reliability of the analysis and to assess the universality of the observed effects, calculations were performed for two different container vessel models that differed significantly in size and principal dimensions (Table 4). The first model represented a medium-size vessel, while the second represented a larger, ocean-going container ship. Two representative container ship hulls available as ready-made models in Maxsurf Modeler were selected for the analysis. This approach made it possible to evaluate the influence of intermediate sections for both a smaller and a much larger hull, while maintaining the same ship type.

**Table 4:** Ship data used for analysis.

Parameter	Container Vessel no1	Container Vessel no2
Lpp [m]-Length between perpendiculars	100,00	198,00
B [m]- Breadth, moulded	18,00	32,21
T [m]- Design draught	6,60	10,56

Conducting the analysis on two ship models makes it possible to demonstrate that the observed influence of intermediate sections:

- is not an effect specific to a single selected hull,
- occurs systematically across different geometric scales,
- has a universal character and can be related to a broader class of ships.

Such an approach strengthens the credibility of the conclusions and reduces the risk of interpreting the results as accidental or dependent solely on a specific geometry.

Container ships constitute a particularly suitable object for analyzing the influence of section discretization because they are characterized by:

- slender and strongly curved bow and stern regions,
- pronounced changes in waterplane sectional area along the hull length,
  - significant sensitivity of stability parameters to the accuracy of calculations of the underwater hull geometry.

Under such conditions, errors resulting from an insufficient number of sections are easier to observe and interpret unambiguously. At the same time, container ships belong to the ship types most frequently analyzed in engineering practice (due to both their large number in operation and the number of stability-related accidents involving this type of vessel), which increases the practical relevance of the presented results.

For each analyzed hull, calculations were performed for three section configurations

#### Basic configuration (21 stations).

**Fig 6:**
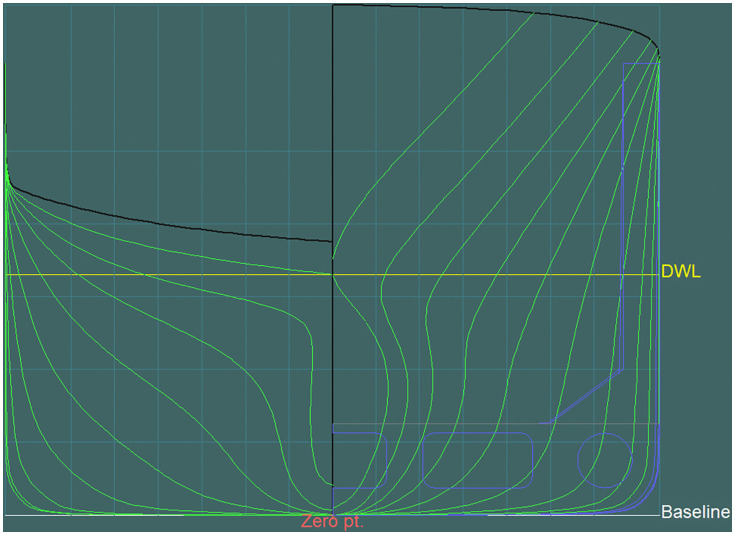
Transverse sections at theoretical frames in the basic configuration (21 stations).

**Fig 7:**
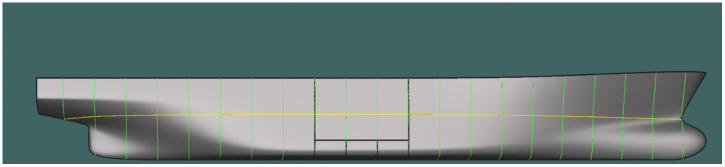
Transverse sections at theoretical frames in the basic configuration – longitudinal hull section (21 stations).

Sections evenly distributed between the forward and aft perpendiculars (Fig 6 and 7).

#### Configuration with intermediate sections (23 stations).

**Fig 8:**
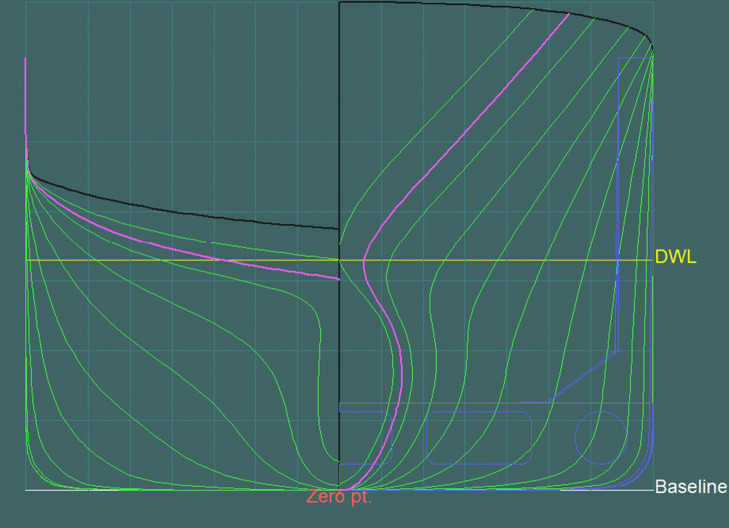
Transverse sections at theoretical frames in the configuration with intermediate sections (23 stations).

**Fig 9:**
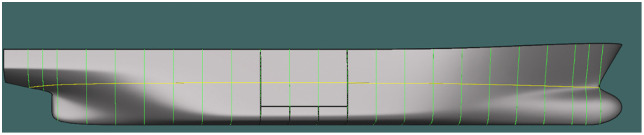
Transverse sections at theoretical frames in the configuration with intermediate sections– longitudinal hull section (23 stations).

Two additional sections were added in the bow and stern regions, halfway between the first and last sections (Fig 8 and 9).

#### Densified configuration (25 stations).

**Fig 10:**
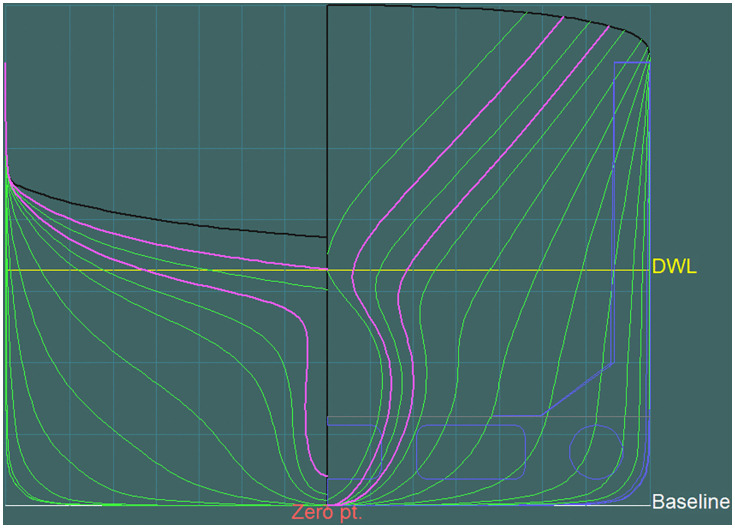
Transverse sections at theoretical frames in the densified configuration (25 stations).

**Fig 11:**
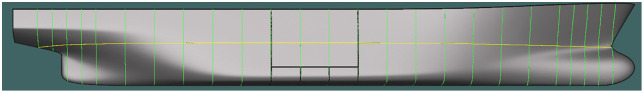
Transverse sections at theoretical frames in the densified configuration – longitudinal hull section (25 stations).

Four additional sections were added in the bow and stern regions to further increase discretization density (Fig 10 and 11).

In each case, the hull geometry remained identical; only the number of sections used in the calculations was changed. The calculation results obtained using the software and ready-made models for selected geometric and stability parameters for the two container vessel models are presented in Tables 5 and 6.

**Table 5:** Results of geometric characteristic calculations for Container Vessel no.1.

No.	Parametr	Results (21 stations)	Results (21 + 2 stations)	Results (21 + 4 stations)
1	Displacement [t]	8015	8004	8003
2	Heel [°]	0,0	0,0	0,0
3	Draft at FP [m]	6,600	6,600	6,600
4	Draft at AP [m]	6,600	6,600	6,600
5	Draft at LCF [m]	6,600	6,600	6,600
6	Trim (+ve by stern) [m]	0,000	0,000	0,000
7	WL Length [m]	99,986	99,986	99,986
8	Wetted Area [m²]	2405,947	2358,314	2359,169
9	Waterpl. Area [m²]	1400,826	1394,648	1395,279
10	Prismatic coeff. (Cp)	0,665	0,664	0,664
11	Block coeff. (Cb)	0,658	0,657	0,657
12	Max Sect. area coeff. (Cm)	0,990	0,990	0,990
13	Waterpl. area coeff. (Cwp)	0,778	0,775	0,775
14	LCB from zero pt. (+ve fwd) [m]	48,719	48,777	48,780
15	LCF from zero pt. (+ve fwd) [m]	45,259	45,451	45,433
16	Z_B_ [m]	3,506	3,506	3,506
17	KG [m]	6,600	6,600	6,600
18	BM_t_ [m]	3,910	3,908	3,910
19	BM_L_ [m]	103,748	102,365	102,480
20	GM_t_ [m]	0,816	0,814	0,815
21	GM_L_ [m]	100,654	99,271	99,386
22	KM_t_ [m]	7,416	7,414	7,415
23	KM_L_ [m]	107,254	105,871	105,986
24	RM Righting Moment [tm]	114,169	113,717	113,845

**Table 6:** Results of geometric characteristic calculations for Container Vessel no.2.

No.	Parametr	Results (21 stations)	Results (21 + 2 stations)	Results (21 + 4 stations)
1	Displacement [t]	36671	36763	36765
2	Heel [°]	0,0	0,0	0,0
3	Draft at FP [m]	10,560	10,560	10,560
4	Draft at AP [m]	10,560	10,560	10,560
5	Draft at LCF [m]	10,560	10,560	10,560
6	Trim (+ve by stern) [m]	0,000	0,000	0,000
7	WL Length [m]	192,890	192,890	192,890
8	Wetted Area [m²]	7132,554	7202,050	7230,876
9	Waterpl. Area [m²]	4713,299	4716,634	4717,180
10	Prismatic coeff. (Cp)	0,578	0,579	0,579
11	Block coeff. (Cb)	0,540	0,541	0,541
12	Max Sect. area coeff. (Cm)	0,944	0,944	0,944
13	Waterpl. area coeff. (Cwp)	0,759	0,759	0,759
14	LCB from zero pt. (+ve fwd) [m]	93,114	93,532	93,507
15	LCF from zero pt. (+ve fwd) [m]	86,164	86,153	86,103
16	ZB [m]	6,049	6,047	6,044
17	KG [m]	10,560	10,560	10,560
18	BMt [m]	9,691	9,655	9,648
19	BML [m]	258,663	258,778	258,686
20	GMt [m]	5,180	5,142	5,133
21	GML [m]	254,152	254,265	254,171
22	KMt [m]	15,740	15,702	15,692
23	KML [m]	264,711	264,825	264,730
24	RM Righting Moment [tm]	3315,428	3299,405	3293,367

Increasing the number of sections from “21” to “21 + 2” and “21 + 4” results in systematic, though relatively small, changes in the calculated hydrostatic parameters. The greatest sensitivity is observed in parameters related to the geometric integration of the underwater hull, primarily the wetted surface area, which decreases by approximately 1.9% (“21 + 2” compared to “21”) and about 1.8% (“21 + 4” compared to “21”). This indicates that the basic variant overestimates local hull curvatures, while section densification leads to a more realistic representation of the surface.

Displacement changes only slightly (−0.15% relative to the basic variant), which means that the global underwater volume is relatively stable; however, its longitudinal distribution is corrected. This is confirmed by a shift of the center of buoyancy (LCB) by approximately +0.06 m and the center of flotation (LCF) by about +0.19 m relative to the “21” variant. Although these values appear small, they are engineering-significant in trim and longitudinal equilibrium analyses.

In the area of stability parameters, small but systematic changes are observed. BMt (transverse metacentric radius) decreases in the “21 + 2” variant, while in “21 + 4” it practically returns to the base value. GMt (initial transverse metacentric height) decreases by approximately 0.25% (“21 + 2” compared to “21”), while the differences between “21 + 2” and “21 + 4” are already minimal. A similar trend is observed for the righting moment RM at 1°, which decreases by approximately 0.4–0.5% compared to the base variant. This means that coarser discretization may lead to a slight overestimation of initial transverse stability.

Increasing the number of sections from “21” to “21 + 2” and “21 + 4” leads to systematic corrections of the calculated hydrostatic parameters, with the greatest sensitivity observed in quantities resulting from geometric integration along the hull length. The most significant changes were noted for the wetted surface area (increase of up to approximately 1.4%) and for the position of the center of buoyancy LCB (shift on the order of 0.4 m). This indicates that with coarser discretization, local curvature variations of the hull are smoothed out, resulting in an underestimation of both wetted surface and underwater volume. Displacement increases by only about 0.25%, confirming that the global volume is relatively stable, while its longitudinal distribution undergoes a measurable correction.

These changes translate directly into stability parameters. The transverse metacentric radius BMt and the initial transverse metacentric height GMt decrease by approximately 0.4–0.9%, and the righting moment at 1° heel decreases by about 0.6%. This means that the variant with fewer sections slightly overestimates initial transverse stability.

The largest differences occur when moving from “21” to “21 + 2” sections, while further densification (“21 + 4”) results only in minor corrections, indicating numerical convergence of the solution. The results clearly show that the accuracy of hydrostatic calculations depends not only on the applied integration scheme but also, to a significant extent, on the manner in which hull geometry is represented.

For both analyzed vessels, consistent and repeatable trends were observed as the number of transverse sections increased:

- the introduction of intermediate sections led to changes in calculated displacement, indicating improved accuracy in determining underwater volume,
  - the position of the center of buoyancy (LCB) shifted along the ship length, confirming the sensitivity of static moments to discretization density,
  - stability parameters, particularly the initial metacentric height GMt and the righting moment RM for small heel angles, underwent small but systematic changes,
  - for both models, clear convergence of results was observed between configurations with 23 and 25 sections, indicating the achievement of a stable numerical solution.

In both cases, the configuration with 21 sections produced results that differed from those obtained with a greater number of sections, confirming that excessively coarse discretization can cause significant numerical integration errors.

Despite similar trends, the scale of the observed changes differed between the analyzed models:

- for the larger container ship, absolute differences in displacement and shifts in LCB were clearly greater than for the smaller vessel,
- the influence of intermediate sections on parameters dependent on waterplane shape (GMt, RM) was more noticeable for the larger hull,
  - the greater ship length increased the sensitivity of calculations to discretization accuracy in the bow and stern regions.

These differences result directly from the fact that as hull length increases, the integration domain and the contribution of geometrically complex regions to static moment calculations also increase. This means that larger ships require relatively denser section grids to achieve comparable calculation accuracy.

The obtained results demonstrated a clear influence of the number of sections on the calculated hydrostatic and stability parameters. In particular, the following were observed:

- an increase in calculated displacement of approximately 0.25% after introducing intermediate sections,
  - a shift in the position of the center of buoyancy (LCB) of approximately 0.4 m,
  - a reduction in metacentric height GMt of approximately 0.7–0.9%.

These changes result from a more accurate representation of hull geometry in the bow and stern regions, which have a significant influence on volume and static moment integration.

The analysis conducted on two container ships with significantly different dimensions confirmed that the method of hull geometry discretization has a measurable impact on the calculated hydrostatic parameters. Increasing the number of intermediate sections leads to systematic corrections of the results, with the magnitude of changes depending on vessel size.

The results clearly indicate that the accuracy of classical hydrostatic calculations depends not only on the applied integration scheme but also, to a significant extent, on the method of hull geometry representation. In the context of modern regulatory requirements and stability analyses under non-steady conditions, the use of densified discretization is fully justified.

## Discussion

The results presented in this study show that accounting for intermediate sections has a measurable and systematic impact on the accuracy of classical hydrostatic calculations. The observed reduction in numerical integration error confirms that the discretization strategy plays a key role in determining underwater volume and center of buoyancy coordinates, even when well-established numerical integration methods are used. This conclusion supports the view that numerical accuracy in hydrostatics depends not only on the choice of integration method but also on how accurately the hull geometry is represented along the integration domain.

Although relative differences in calculated hydrostatic parameters may appear small, their practical significance should be considered in the context of stability analyses. Errors in determining underwater volume and center of buoyancy position directly affect derived quantities such as metacentric height and righting arm characteristics. In particular, longitudinal errors in the position of the center of buoyancy can influence trim-related stability assessment, while vertical errors propagate into the evaluation of initial stability. As modern stability regulations increasingly require hydrostatic analyses under conditions corresponding to the ship being on a wave crest or in a wave trough (second-generation intact stability criteria, SGISC), the sensitivity of stability predictions to errors resulting from geometric discretization becomes even more important.

From an engineering perspective, the results indicate limitations of the uniform hull subdivision strategy commonly used in routine hydrostatic analyses. The presented case study shows that omitting intermediate sections in areas of high geometric variability, such as the bow and stern, may lead to accumulation of numerical errors. Selective introduction of intermediate sections in these areas significantly improves accuracy without a proportional increase in computational effort. This suggests that adaptive discretization strategies or approaches that account for local geometric features can provide a more effective balance between accuracy and computational cost than uniform subdivision methods.

The obtained results are consistent with observations reported in related fields of engineering analysis, where numerical results are known to strongly depend on discretization strategy and geometric representation accuracy. Similar sensitivity has been demonstrated in structural and hydrodynamic modeling, as well as in analyses of irregular geometries in other engineering disciplines. In a broader context, the results of this study highlight the importance of controlling discretization-related errors as a necessary condition for obtaining reliable and repeatable numerical results.

A proposed optimization scheme based on a genetic algorithm is discussed as a potential extension of this work. Its intended purpose is to support automatic selection of the number and distribution of intermediate sections, as well as numerical integration strategies, based on local geometric features and specified accuracy requirements. However, the genetic algorithm scheme was not implemented or validated in this study and should be treated solely as a direction for future research. Comprehensive evaluation of such an approach would require dedicated numerical experiments, comparisons with standard discretization strategies, sensitivity analyses of algorithm parameters, and assessment of computational cost.

Certain limitations of this study should also be noted. The numerical analysis is based on a selected ship case; therefore, the results should be treated as illustrative rather than universal. Moreover, the influence of discretization errors on global stability characteristics was discussed qualitatively rather than through direct stability simulations. Future research should include a wider range of hull forms, incorporate direct coupling with stability calculations, and evaluate the effectiveness of adaptive or optimization-based discretization strategies across a broader range of operational scenarios.

In summary, the presented discussion confirms that intermediate sections constitute an important, though often underestimated, factor in classical hydrostatic calculations. By quantitatively demonstrating their influence on numerical accuracy, this study provides a solid basis for improving discretization practices in engineering hydrostatics and establishes a foundation for further research into automated and adaptive methods aimed at increasing the accuracy of hull geometry analyses.

## Conclusions

This study quantitatively investigated the influence of incorporating intermediate sections on the accuracy of geometric and hydrostatic calculations of a ship hull. The results demonstrate that the inclusion of intermediate sections leads to a systematic reduction of numerical integration errors in the estimation of submerged volume and center-of-buoyancy coordinates. Although the absolute differences may appear small in relative terms, the observed error reduction is non-negligible from an engineering perspective, particularly when such parameters are subsequently used in stability-related assessments.

The comparative analysis of selected classical numerical integration schemes confirms that discretization strategy plays a decisive role in the accuracy of hydrostatic calculations. Introducing intermediate sections in regions of pronounced geometric variability significantly improves the fidelity of the results without requiring excessive increases in computational complexity. This finding highlights the importance of adapting hull discretization to local geometric characteristics rather than relying solely on uniform subdivision strategies.

The presented case study illustrates that neglecting intermediate sections may lead to cumulative numerical inaccuracies that affect key hydrostatic parameters. Such inaccuracies may propagate into stability calculations, potentially influencing the reliability of safety-related evaluations, especially under conditions where hydrostatic properties are assessed outside equilibrium states. The results therefore support the need for careful control of discretization schemes in practical hydrostatic analyses and engineering applications.

Based on the findings of this study, a conceptual framework for the application of genetic algorithms to the optimization of hull discretization is proposed as a direction for future research. The intended role of this framework is to support the automated selection of the number and spatial distribution of intermediate sections, as well as the numerical integration strategy, while balancing computational cost and accuracy. The genetic algorithm approach is not implemented or validated within the present work and is explicitly identified as future work requiring dedicated numerical experiments, benchmarking against conventional approaches, and sensitivity analysis.

In summary, this study provides empirical evidence of the importance of intermediate sections in classical hydrostatic calculations and demonstrates their measurable impact on numerical accuracy. The results contribute to improved understanding of discretization-related errors in hull geometry analysis and establish a methodological basis for further research on automated and adaptive discretization strategies aimed at enhancing the robustness and reproducibility of hydrostatic calculations in naval engineering.

## Supporting information

S1 File Appendix A. Detailed computational example for evaluation of numerical method accuracy.
(DOCX)
