# Supplementary material for: Analysis of the importance of using intermediate intervals in the calculation of geometrical characteristics of the ship’s hull
Source: PLoS One. 2026 May 8;21(5):e0348523. doi: 10.1371/journal.pone.0348523 (PMC13155633; doi:10.1371/journal.pone.0348523)
Supplement: S1 File — Detailed computational example for evaluation of numerical method accuracy. (DOCX) [file pone.0348523.s001.docx]

**APPENDIX A**

**Numerical Integration Methods**

To demonstrate the principles of approximate integration and to assess their accuracy, a definite integral of a known function (Fig. A1) is solved as an example:

$$f\left( x \right)=x^{2}+4, <x_{i}=1,x_{f}=5>$$

(A1)

$$\int_{1}^{5} x^{2}+4dx=\int x^{2}dx+4\int x^{0}dx=\frac{x^{2}}{3}+4x$$

(A2)


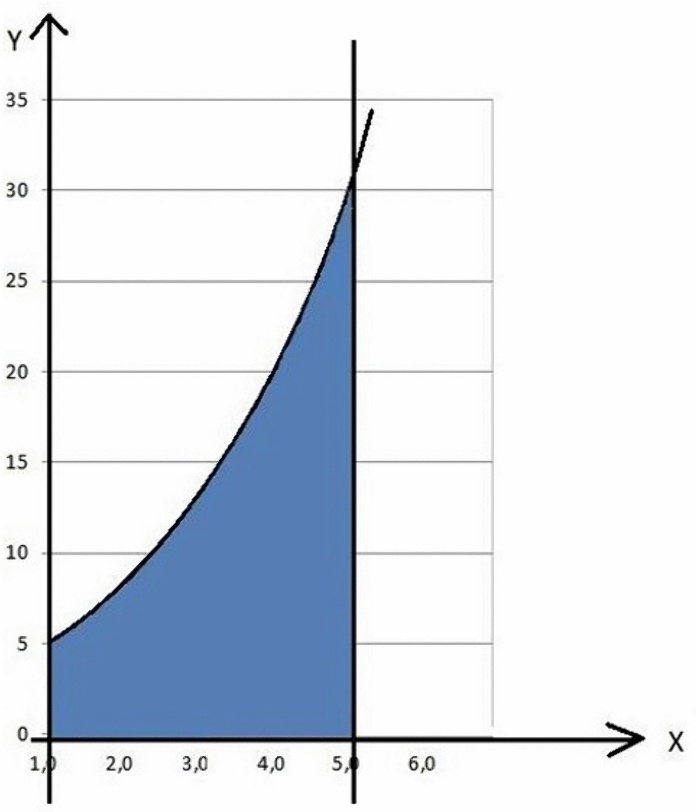


**Fig. A1. Plot of the function f(x) over the interval ⟨x_i_=1, x_f_=5⟩**

Solving the above equation over the interval *<x_i_=1, x_f_=5>* the resulting area under the curve of the analyzed function is A=57,333… [u^2^] (u - denotes the unit).

**Rectangle Method (Midpoint Rule)**

When choosing to apply an approximate integration method using rectangles, the procedure is as follows:

The integration interval *⟨i, f⟩* is divided into *n* equal subintervals, with the understanding that the accuracy of the result increases with the number of subdivisions. The assumed number of subintervals *n* allows the determination of a step size *d*, which represents the distance between successive points. In the given example, the number of subintervals is assumed to be *n=4* . Then, under the curve of the function over the interval, rectangles are fitted appropriately depending on the chosen rectangle integration method. The sides of the rectangles are defined as follows: the width *d* (the base of each rectangle), and the height *y*, which is the value of the function *f(x)* at a selected point within the subinterval. This value is either computed or measured at the chosen evaluation point.

The value of each rectangle depends on the specific variation of the rectangle method (e.g., left endpoint, right endpoint, or midpoint). The final result of the numerical integration is the sum of the products of the subinterval width *d* and the function value *y* at each corresponding point *x*.

The general formula is expressed as follows:

$$\int_{xi}^{xf} f\left( x \right)=\sum_{i=1}^{n} f(x_{i})\cdot dx$$

(A3)

**The following rectangle-based integration methods are distinguished:**

- **Left-hand Rule,**
- **Right-hand Rule,**
- **Midpoint Rule.**

**Left-hand Rule Method:**

This method is characterized by taking the evaluation point for the first rectangle at position *p*, i.e., at the lower limit of integration.

The general formula for the approximate value of the definite integral using this method is:

$$A_{L}=\sum_{i=1}^{n} f(X_{i-1})\cdot dx$$

(A4)

For the function analysed over the specified interval, the area computed using the left-hand rectangle method is *A_L_*=46 [u^2^].

Right-hand Rule Method:

In the case of the right-hand rectangle method, the function value *y* for the first rectangle is calculated at the point *x_i_+ Δx,* i.e., the lower bound of integration increased by the step size *Δx*.

The general formula for the approximate value of a definite integral using this method is:

$$A_{R}=\sum_{i=1}^{n} f(X_{i})\cdot dx$$

(A5)

For the analyzed function over the specified interval, the area computed using the right-hand rectangle method is *A_R_*=70 [u^2^].

Midpoint Rule:

When applying the midpoint rule, the point *x_1_* of the first rectangle is taken at the midpoint between the lower limit of integration *x_i_* and the point increased by the value *Δx.*

$$A_{M}=\sum_{i=1}^{n} f(\frac{(X_{i-1}+x_{i})}{2})\cdot dx$$

(A6)

For the analyzed function over the specified interval, the area computed using the right-hand rectangle method is *A_M_*=57 [u^2^].

**Trapezoidal Rule**

Analogous to the previous method, the operations for approximating definite integrals using the trapezoidal rule begin by dividing the area under the function curve, bounded by the interval *⟨i,f⟩* into *n* equal subintervals.

The general formula for the approximate integral using the trapezoidal rule is:

$$A_{T}=\frac{X_{f}-x_{i}}{2\cdot n} [f(x_{i}+2\left( \sum_{i=1}^{n-1} (f+i\cdot dx) \right)+f\left( x_{f} \right)]$$

(A7)

**For the analyzed function over the given interval, the area calculated using the trapezoidal rule is** *A_T_*=57 [u^2^].

**Simpson’s Rule**

Simpson’s Rule is a slightly different technique for obtaining an approximate value of a definite integral. In the previous two methods, the result of integration was approximated by the sum of areas of individual figures into which the region under the graph of the function *y=f(x)* was divided. In Simpson’s Rule, the approximation is not performed directly under the actual graph of the integrated function *y=f(x).* Instead, the result of integration is obtained by summing the areas under a set of parabolic functions *y=P(x):*

$$\int_{x_{i}}^{x_{f}} f(x)dx=\int_{x_{i}}^{x_{f}} P(x)dx=\int_{x_{i}}^{x_{f}} ax^{2}+bx+c\cdot dx$$

(A8)

The general formula for the approximate integral using Simpson’s Rule is given by the equation:

$$A_{S}=\frac{dx}{3}\cdot(4f\left( x_{1} \right)+2f\left( x_{2} \right)+\ldots+2f\left( x_{n-2} \right)+4f(x_{n-1)}+f\left( x_{n} \right))$$

(A9)

**For the analysed function over the given interval, the area calculated using Simpson’s rule is** *A_S_*=57,333 [u^2^].

**Table A1. Summary of Results from Presented Numerical Integration Methods**

| **Method** | **Number of intervals** | **Results** | **Accuracy [%]** |
| --- | --- | --- | --- |
| **Definite integral** | ∞ | 57,333… | 100 |
| **Left-hand rule** | 4 | 46 | 80,2 |
|  | 100 | 56,8544 | 99,165 |
|  | 1000 | 57,28534 | 99,91629 |
| **Right-hand Rule** | 4 | 70 | 77,90 |
|  | 100 | 57,8144 | 99,161 |
|  | 1000 | 57,38134 | 99,91627 |
| **Midpoint Rule** | 4 | 57 | 99,4 |
|  | 10 | 57,28 | 99,907 |
|  | 100 | 57,3328 | 99,99907 |
|  | 1000 | 57,333328 | 99,999999 |
| **Trapezoidal Rule** | 4 | 58 | 98,80 |
|  | 10 | 57,44 | 99,814 |
|  | 100 | 57,3344 | 99,99814 |
|  | 1000 | 57,333344 | 99,99998 |
| **Simpson’s Rule** | 2 | 57,333… | 100 |
|  | 6 | 57,333… | 100 |

**The summary of the of the definite integral solution for the analyzed function** *f(x)=x_2_+4*, over the interval *⟨x_p_=1,x_k_=5⟩,* using various numerical integration methods, along with their accuracy in relation to the reference (exact) result depending on the number of created subintervals *n* **results is presented in Table A1**.

To obtain the numerical integration result for 𝑛 =1000 subintervals—while reducing computational effort and minimizing the risk of error—a custom computational algorithm was developed. This algorithm, implemented on a general-purpose computational platform, enabled the calculation of the numerical integral for each of the described methods, regardless of the number of subintervals *n* in the integration domain.

When interpreting the summarized results, particular attention should be given to the relationship between the number of subintervals n and the final result’s accuracy. Table 1 clearly shows a significant increase in accuracy with an increasing number of subintervals. By understanding how the number of subdivisions *n* affects result precision, one can choose the optimal number of intervals according to the needs of a given application.

**Simpson’s Rule** yielded **100% accuracy** for the chosen function and proved to be the most accurate among the tested methods. In the examined example, the **Mid-point Rule** ranks second in accuracy. However, despite using *n=1000* subintervals, it did not reach the *100%* accuracy achieved by Simpson’s method with only *n=2* intervals.

**The Trapezoidal Rule** produced results nearly as accurate as the Mid-point Rule. At the same level of subdivision, the similarity in accuracy allows these two methods to be used interchangeably. It must be noted, however, that for other functions, the accuracy ranking between these methods may vary, but both are expected to remain in the upper tier—right below Simpson’s Rule.

The **Left-hand** and **Right-hand Rectangle Methods** showed the **highest error**. These techniques are suitable in cases where high precision is not required. The results in Table 1 indicate that to reach an accuracy comparable to the other methods, one would need to use **100 times more subdivisions**, leading to significantly higher computational cost to achieve the desired result.
